# Supplementary material for: Olympic Cycle Comparison of the Nutritional and Cardiovascular Health Status of an Elite-Level Female Swimmer: Case Study Report from Slovenia
Source: Sports (Basel). 2022 Apr 20;10(5):63. doi: 10.3390/sports10050063 (PMC9143311; doi:10.3390/sports10050063)
Supplement: Supplementary file 1 [file sports-10-00063-s001.zip › sports-1632304-supplementary.pdf]

Table S1. The athlete's segmental LST.

Parameter	Year 2018	Year 2022
LST segmental (kg)		
LST left arm	2.55	**2.78**
LST right arm	2.40	**2.91**
LST trunk	22.4	**24.8**
LST left leg	7.45	**8.36**
LST right leg	7.29	7.74
LST head	2.85	3.04

Significant change in value is written bold (i.e., ≥9% of relative change). LST: lean soft tissue.
